# Supplementary material for: Mycobacterium tuberculosis YrbE3A Promotes Host Innate Immune Response by Targeting NF-κB/JNK Signaling
Source: Microorganisms. 2020 Apr 17;8(4):584. doi: 10.3390/microorganisms8040584 (PMC7232258; doi:10.3390/microorganisms8040584)
Supplement: Supplementary file 1 [file microorganisms-08-00584-s001.pdf]

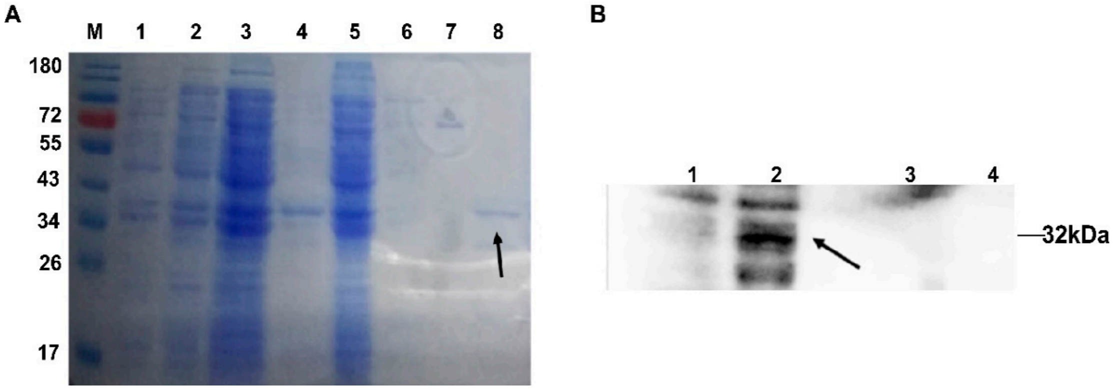

Figure S1. Identification of YrbE3A expression and cell location. (**A**) SDS-PAGE (12%) was used to determine the expression of the recombinant YrbE3A protein Lane M, PageRuler Prestained Protein Ladder; Lane 1, total Ms_YrbE3A cell lysate without heat-inducible; Lane 2, total Ms_YrbE3A cell lysate with heat-inducible; Lane 3, proteins in the supernatant; Lane 4, proteins in the pellet; Lane 5, binding buffer after washing; Lanes 6,7, washing buffer after washing; Lane 8, the purified YrbE3A protein. Arrow indicates the YrbE3A protein of 32 kDa. (**B**) Western blot assay on location of YrbE3A protein. The cellular proteins in cell lysate and bacterial membrane of Ms_YrbE3A and Ms_vec in SDS-PAGE were blotted to PVDF membrane. The blot was probed with His-tag monoclonal antibody. Lane 1, YrbE3A-cytoplasm; lane 2, YrbE3A-cytomembrane; Lane 3, Vec-cytoplasm; line 4, Vec-cytomembrane. Arrow indicates the YrbE3A protein.

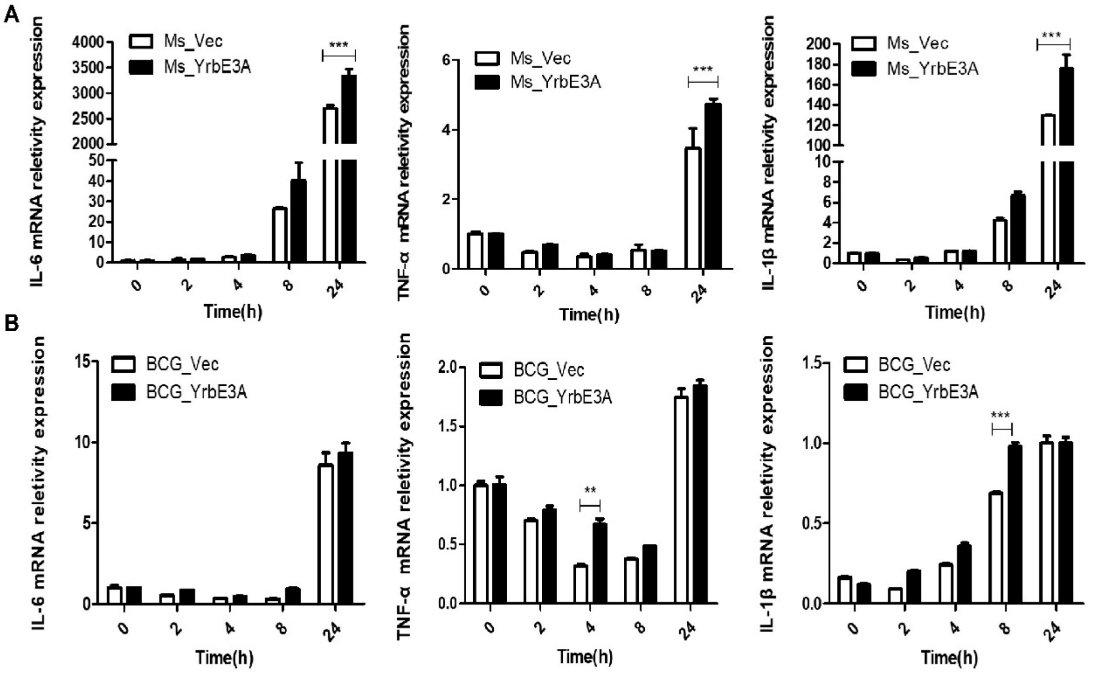

Figure S2. Transcription of cytokine expression of Ms_YrbE3A, Ms_Vec, BCG_YrbE3A, and BCG_Vec in infected RAW264.7 cells. (A,B) Quantitative RT-PCR was used to determine transcription levels of IL-6, TNF-α, and IL-1β by RAW264.7 infected by Ms_YrbE3A and Ms_Vec (A.). BCG_YrbE3A and BCG_Vec (B). Significant differences (*$p < 0.05$, **$p < 0.01$, ***$p < 0.001$) among each treatment were analyzed by a two-way ANOVA followed by the LSD test. Each sample was repeated in triplicate.

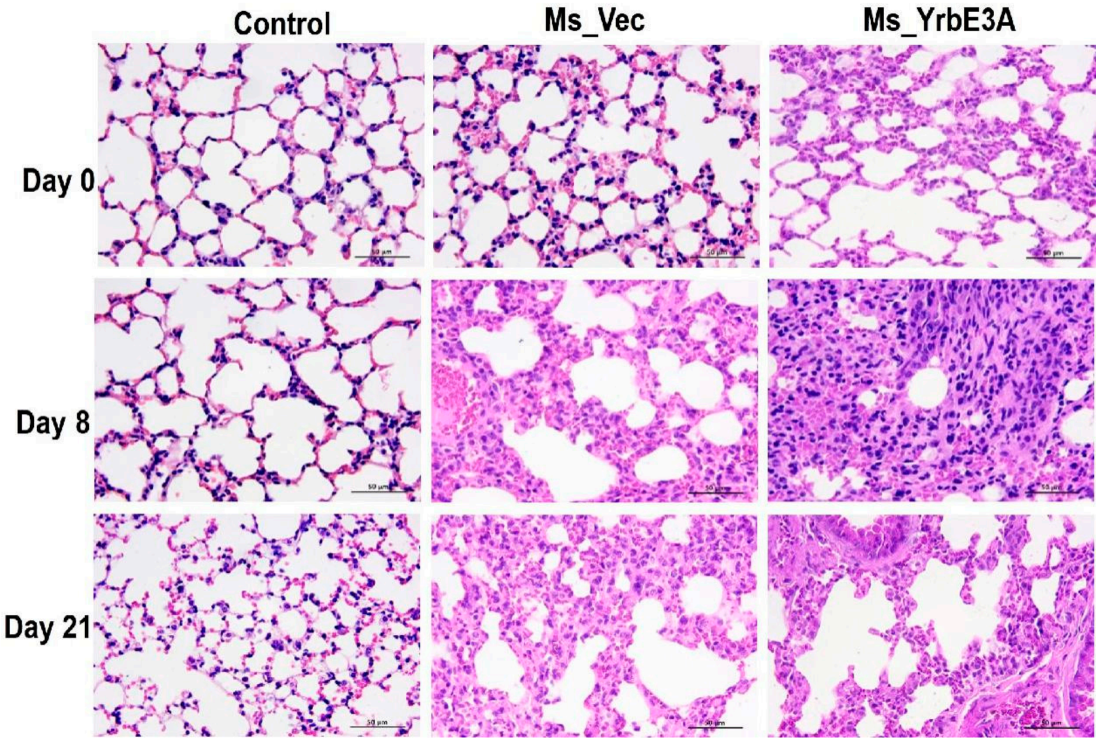

Figure S3. YrbE3A expression promoted lung inflammatory lesions in mice infected with recombinant Ms_YrbE3A and Ms_Vec. Mice were infected with Ms_YrbE3A and Ms_Vec at a dose of 2.0×10^6 CFU via intratracheal challenge, euthanized at day 0, 8, and 21 postinfection, and lung tissues were observed after being sectioned and stained with Hematoxylin and eosin (H&E) stain. Scale bar: 50 μm. Data are representative of one experiment with two independent biological replicates (mean ± SEM of n = 5 mice per group).

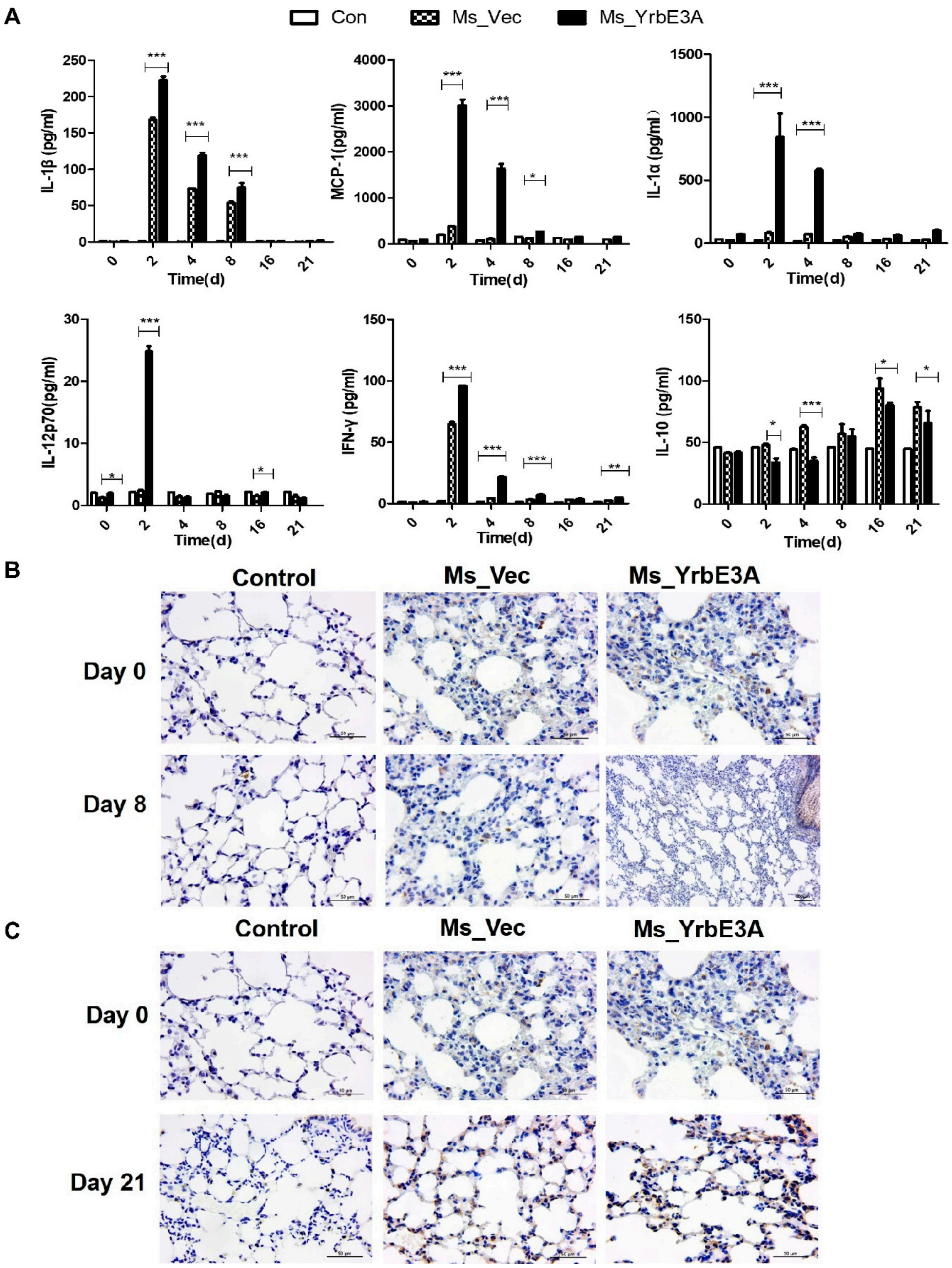

Figure S4. Detection of lung inflammatory cytokines in Ms_YrbE3A- and Ms_Vec-infected C57BL/6 mice. (A) Multiplex immunoassay of IL-1β, MCP-1, IL-1*α*, IL-12p70, IFN-*γ*, and IL-10 in lung cells from C57BL/6 mice infected intratracheally for 0–21 d with 2.0 × 10^6 CFU of MS_Vec and MS_YrbE3A strains, while a saline control was used as the placebo. (B,C) The detection of the cytokines IL-6 (left panel) and TNF-*α* (right panel) in the lungs of experimental mice by immunohistochemistry at labelled days postinfection. Significant difference (*$p < 0.05$, **$p < 0.01$, ***$p < 0.001$) among treatments was analyzed by a two-way ANOVA followed by the LSD test. Each sample was repeated in triplicate.
